# A Nasal Epithelial Receptor for *Staphylococcus aureus* WTA Governs Adhesion to Epithelial Cells and Modulates Nasal Colonization

**DOI:** 10.1371/journal.ppat.1004089

**Published:** 2014-05-01

**Authors:** Stefanie Baur, Maren Rautenberg, Manuela Faulstich, Timo Grau, Yannik Severin, Clemens Unger, Wolfgang H. Hoffmann, Thomas Rudel, Ingo B. Autenrieth, Christopher Weidenmaier

**Affiliations:** 1 Interfacultary Institute for Microbiology and Infection Medicine Tübingen, Department of Medical Microbiology and Hygiene, University of Tübingen, Tübingen, Germany; 2 Lehrstuhl für Mikrobiologie, Biozentrum der Universität Würzburg, Würzburg, Germany; 3 Institute for Tropical Medicine, University of Tübingen, Tübingen, Germany; 4 German Center for Infection Research (DZIF), Partner Site Tübingen, Tübingen, Germany; Harvard Medical School, United States of America

## Abstract

Nasal colonization is a major risk factor for *S. aureus* infections. The mechanisms responsible for colonization are still not well understood and involve several factors on the host and the bacterial side. One key factor is the cell wall teichoic acid (WTA) of *S. aureus*, which governs direct interactions with nasal epithelial surfaces. We report here the first receptor for the cell wall glycopolymer WTA on nasal epithelial cells. In several assay systems this type F-scavenger receptor, termed SREC-I, bound WTA in a charge dependent manner and mediated adhesion to nasal epithelial cells *in vitro*. The impact of WTA and SREC-I interaction on epithelial adhesion was especially pronounced under shear stress, which resembles the conditions found in the nasal cavity. Most importantly, we demonstrate here a key role of the WTA-receptor interaction in a cotton rat model of nasal colonization. When we inhibited WTA mediated adhesion with a SREC-I antibody, nasal colonization in the animal model was strongly reduced at the early onset of colonization. More importantly, colonization stayed low over an extended period of 6 days. Therefore we propose targeting of this glycopolymer-receptor interaction as a novel strategy to prevent or control *S. aureus* nasal colonization.

## Author Summary

About 20% of the human population is colonized by *Staphylococcus aureus*. The reservoir of *S. aureus* is mainly the human nose. Usually, colonization does not lead to infection and is therefore without symptoms. However, when hospitalized patients exhibit a suppressed immune system, they are at risk of getting infected by their own nasal *S. aureus* strain. Therefore, it is important to understand the events and mechanisms underlying colonization. Until now *S. aureus* nasal colonization is only partially understood. One bacterial key factor is a sugar polymer of *S. aureus*, termed cell wall teichoic acid (WTA), which is involved in *S. aureus* adhesion to cellular surfaces in the inner part of the nasal cavity. We show here that a receptor-protein, which is expressed on such cells, binds WTA and is thereby involved in adhesion of *S. aureus* to nasal cells. This mechanism has a strong impact on nasal colonization in an animal model that resembles the situation in the human nose. Most importantly, inhibition of WTA mediated adhesion strongly reduces nasal colonization in the animal model. Therefore we propose that targeting of this glycopolymer-receptor interaction could serve as a novel strategy to control *S. aureus* nasal colonization.

## Introduction

The nasal cavity is the major reservoir of *Staphylococcus aureus*, which asymptomatically colonizes the anterior nares of 20% of the normal human population persistently [1], [2]. The reasons for the underlying predisposition and the tropism for the human nose are still unclear. If carriers are infected with *S. aureus*, the strain found in the nose is usually also responsible for the infection [1], [2]. Since *S. aureus* is able to cause a variety of severe diseases, the carrier status is an important risk factor in both the community and the healthcare system [1], [3].

Despite the importance of nasal colonization, its molecular basis has still remained largely elusive. Some studies showed that *S. aureus* colonizes mostly the anterior parts of the nares and interacts very efficiently with squamous, keratinized cells [4]. However, there is clear evidence that *S. aureus* also interacts with living ciliated cells in deeper areas of the nasal cavity or even the throat [5], [6], [7], [8] and might be equally abundant in all parts of the nasal cavity [9], [10]. In addition, *S. aureus* is even able to persist intracellularly in nasal epithelial cells of patients suffering from recurrent sinusitis [5].

*S. aureus* nasal colonization is a multifactorial process [11] and host factors [12], as well as *S. aureus* factors like the polysaccharide capsule [13], an array of surface protein adhesins [4], [14], [15], [16], [17], and cell wall teichoic acids (WTA) [6], [18], [19] have been implicated in nasal colonization. One of the important surface protein adhesins with a role is nasal colonization is the cell wall anchored clumping factor B (ClfB). It binds to cytokeratin and keratinized nasal cells [4] and the squamous cell envelope protein loricrin. The impact of this molecular interaction on nasal colonization, has been demonstrated in a mouse model [20].

In a “state of the art” cotton rat model of nasal colonization, protein adhesins of *S. aureus* mainly impact the late stages of nasal colonization whereas the non-protein adhesin WTA played a key role in the initial stages [6]. This is in line with the fact that expression of WTA biosynthesis genes is high during early and later stages of experimental colonization, whereas expression of protein adhesins like ClfB is low in the early stages but high in the late stages of colonization [21], [22]. WTA is a surface-exposed polyanionic cell wall glycopolymer (CWG) composed of about 40 ribitolphosphate repeating units which are modified with D-alanine and N-acetylglucosamine and covalently linked to the peptidoglycan [23], [24] (Figure S1). Several lines of evidence indicate a direct interaction of WTA with receptors on nasal epithelial cells [6], [18]. *In vivo*, WTA is a key factor of nasal colonization since a WTA-deficient *S. aureus* mutant was severely abrogated in colonizing cotton rat noses [18]. Therefore, it is of great importance to understand the molecular basis of WTA-mediated adhesion to the epithelial lining of the inner nasal cavity.

We introduce here SREC-I as a receptor for WTA on nasal epithelial cells. SREC-I, a type F scavenger receptor with six extracellular EGF-like domains, has first been detected on endothelial cells, where the receptor is responsible for the uptake of calreticulin [25] and acetylated low density lipoproteins [26]. Recently, expression of SREC-I was also described in several epithelial cell lines, such as END1, HELA, and Chang [27] epithelial cells. In addition expression, albeit at low levels, was also detected in primary human bronchial epithelial cells (HBEC) [28]. We present here the first evidence for a key role of SREC-I in the early phases of *S. aureus* colonization and thus a novel target for decolonization strategies that can possibly help to protect individuals from *S. aureus* infections.

## Results

### SREC-I is expressed on nasal epithelial cells

In differential pull down experiments of solubilized membrane proteins from epithelial cells with cell wall preparations from wild-type *S. aureus*, a mutant without WTA (*tagO*) and a mutant with structurally altered (negatively charged) WTA (*dltA*), SREC-I was found as a potential WTA binding partner (data not shown). To confirm SREC-I expression in primary nasal epithelia of human and cotton rat origin we isolated cotton rat primary epithelial cells (CRNECs) (Figure S2A) and used primary human nasal epithelial cells (HNECs) (Promocell). We evaluated the presence of SREC-I by reverse transcriptase PCR (RT-PCR), FACS analysis and confocal microscopy in these cells.

**Figure 1:**
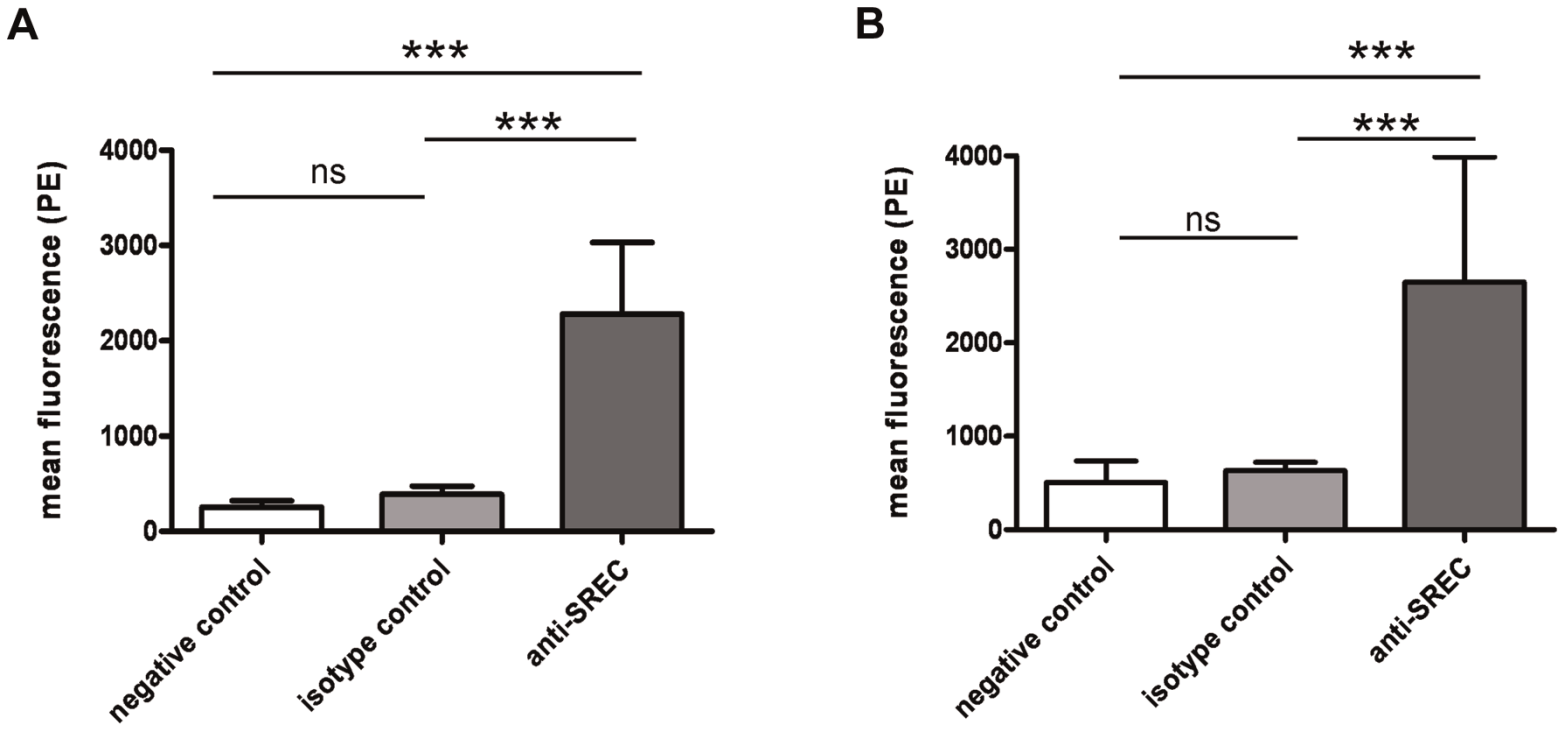
SREC-I on nasal epithelila cells. Surface exposure of SREC-I on HNECs and CRNECs was monitored by FACS with the same anti-human SREC-I antibody and isotype control. Statistical evaluation on HNECs (A) and on CRNECs (B). Data represent means ± SD of 3 or 4 independent experiments. Statistical analysis was performed by Student's t-test. Significant differences are indicated by one (*P*<0.05), two (*P*<0.01), or three (*P*<0.001) asterisks (*).

For detection of SREC-I by FACS analysis we used an anti-human SREC-I antibody and appropriate isotype controls. Both HNECs and CRNECs stained positive for the receptor with this antibody (Figure S2B and Figure 1). RT-PCR Primers were designed based on the mouse and human genome sequence for SREC-I. For both cell types SREC-I mRNA expression could be detected (Figure S2C). Confocal microscopy, employing the same primary antibodies used in the FACS analysis, revealed a weak but specific signal for SREC-I on HNECs and CRNECs (Figure S3). We therefore conclude that SREC-I shows considerable surface exposition on both CRNECs and HNECs.

### SREC-I directly interacts with WTA

**Figure 2:**
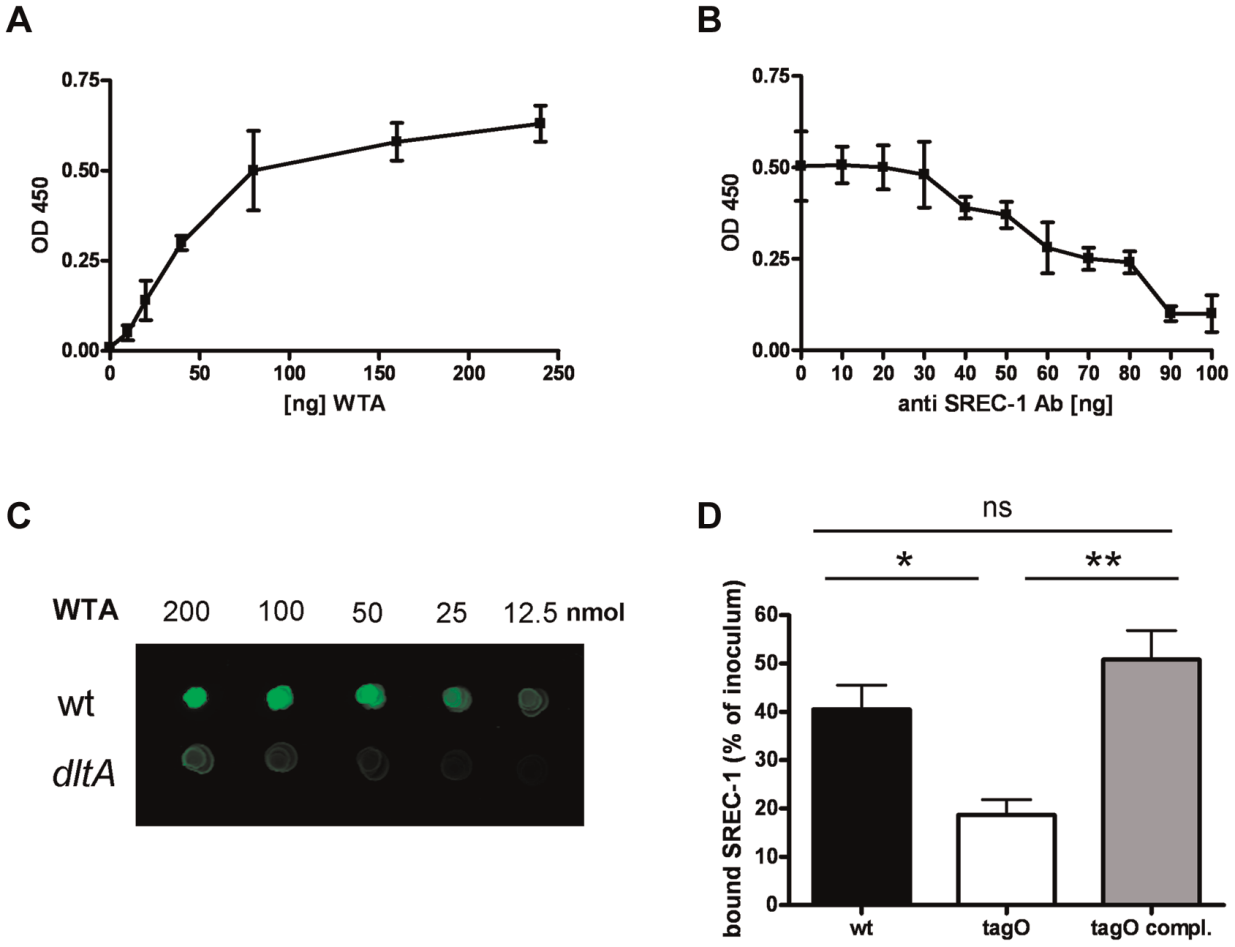
Direct interaction of SREC-I and WTA. Binding of SREC-I to WTA in a microtiter plate-based assay with immobilized WTA at different concentrations. Interaction of a biotin-labeled SREC-I Fc-chimera with WTA was detected with a Streptavidin-HRP conjugate. The data represent means ± SD of 3 independent experiments. (A). Incubation with increasing concentrations of an SREC-I function blocking antibody at a concentration of 80 µg/ml immobilized WTA. The data represent means ± SD of 3 independent experiments. (B).Interaction of WTA spotted in increasing concentrations on a nitrocellulose membrane with a SREC-I Fc-chimera (500 ng/ml)(C). Bound SREC-I Fc-chimera was detected with a secondary antibody coupled to an 800 nm emitting infrared dye on a LI-COR Odyssey infrared imaging system. A representative experiment is shown here (C). Binding of SREC-I to whole bacterial cells, measured with a FITC-labeled SREC-I Fc-chimera. Wild-type *S. aureus*, the isogenic *tagO* mutant and the complemented mutant (*tagO*-pRB*tagO*) were incubated with 50 µg/ml FITC-labeled SREC-I. The bound fluorescence was calculated by subtracting the unbound fluorescence from the total fluorescence of the FITC-labeled SREC-I solution. The mean and SD of 3 independent experiments are shown. Statistical analysis was performed by Student's t-test. Significant differences vs. wild-type strains are indicated by one (*P*<0.05), two (*P*<0.01), or three (*P*<0.001) asterisks (*) (D).

To demonstrate a direct molecular interaction of SREC-I and WTA we employed several experimental setups. Due to the chemical nature of WTA (loss of D-alanine esters during covalent coupling) it was not possible to use a surface plasmon resonance (SPR) based approach on the Biacore platform (data not shown). Therefore, we first studied SREC-I-WTA interaction in a microtiterplate based binding assay. A biotin-labeled soluble human SREC-I Fc-chimera, containing the entire extracellular domains of SREC-I, bound in a dose-dependent and saturable manner to immobilized WTA (Figure 2A). Using a function-blocking SREC-I antibody we were able to block interaction of WTA with SREC-I in a dose-dependent manner (Figure 2B). In addition, we detected a dose dependent interaction of SREC-I with WTA in a ligand blot assay (Figure 2C and Figure S4). In this assay system binding of an SREC-I Fc-chimera to immobilized WTA is detected with the help of an infrared dye-labeled anti-Fc antibody. The SREC-I Fc-chimera bound wild-type (wt) WTA significantly better than negatively charged WTA isolated from a *dlt*A mutant. Furthermore, we directly labeled the SREC-I Fc-chimera with FITC and measured binding of the conjugate to whole bacterial cells in suspension (Figure 2D). We found that wild-type *S. aureus* (wt) bound 41%±12 of the SREC-I in solution whereas a mutant lacking all WTA (*tagO*) bound only 19%±8. The complemented *tagO* mutant bound 51%±15. When we added MgCl_2_ in different concentrations, we could partially decrease the interaction of wild-type *S. aureus* with the FITC labeled SREC-I Fc-chimera to the level of the *tagO* mutant which lacks all WTA. The diminished interaction of the *dltA* mutant, which exhibits negatively charged WTA, was partially restored to wild-type levels at high MgCl_2_ concentrations. In addition we could decrease the interaction of wild-type *S. aureus* to the FITC labeled SREC-I Fc-chimera with WTA purified from wild-type *S. aureus* but not with WTA purified from the *dltA* mutant (Figure S5A). To test the specificity of WTA/SREC-I interaction we used another FITC labeled scavenger receptor in this assay system. The CD36 FITC conjugate showed considerable binding to wild-type *S. aureus*, however we could not detect a considerable decrease in binding of the CD36 FITC conjugate to the *tagO* and *dltA* mutants as we could detect with the FITC labelled SREC-I Fc-chimera (Figure S5B).

### SREC-I mediates WTA-dependent adhesion to nasal epithelial cells

**Figure 3:**
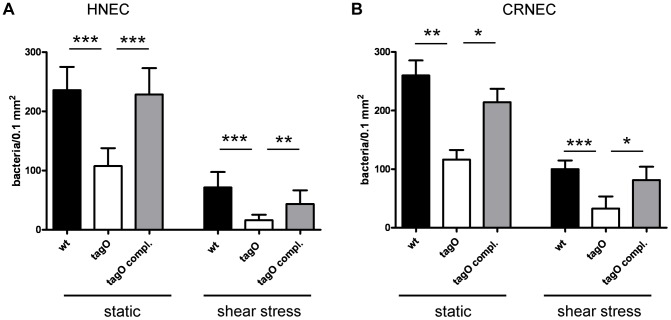
WTA-mediated adhesion of *S. aureus* to HNECs and CRNECs. *S. aureus* adherence to confluent human nasal epithelial cells (HNECs) monitored under static and mild shear stress condition (0.5 dynes) in 24 well plates or flow chamber assays, respectively. Attachment of the *S. aureus* wild-type (wt), *tagO*, and a complemented *tagO* mutant (*tagO* compl.) evaluated microscopically by counting at least 6 visible fields per well or chamber slide containing HNECs (A) or CRNECs (B). The data is expressed as means and SEM of 5 independent experiments (static) or of 6 independent experiments (flow). Statistical analysis was performed by one-way ANOVA with Bonferroni's multiple comparison test. Significant differences between groups are indicated by one (*P*<0.05), two (*P*<0.01), or three (*P*<0.001) asterisks (*).

**Figure 4:**
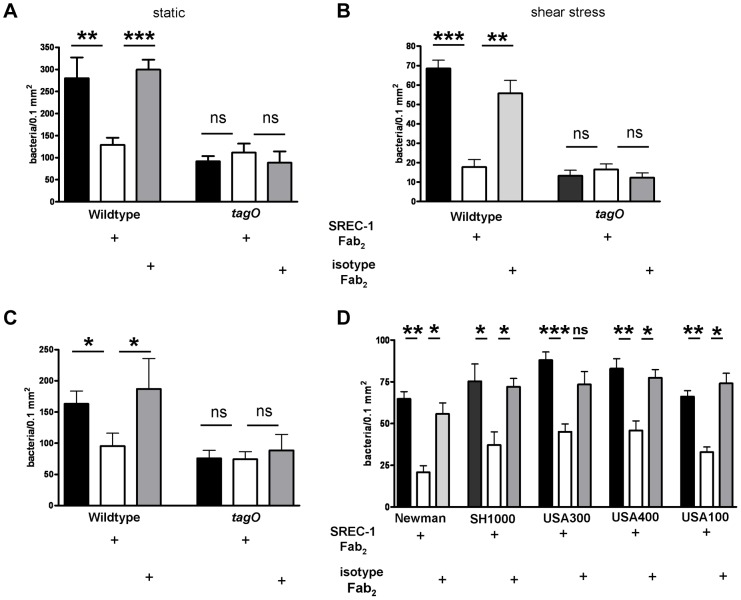
Impact of SREC-I on adhesion of *S. aureus* to HNECs. Inhibition of SREC-I dependent adhesion to HNECS by Fab_2_- fragments of an anti-human SREC-I antibody in 24 well microtiter plate assays under static conditions (A) and shear stress conditions in ibidi chamber slide assays (B). 10 µg/ml of the anti-SREC-I Fab_2_-fragments and isotype control were used in all assays. Attachment of the *S. aureus* wild-type (wt), and *tagO* mutant was evaluated microscopically by counting at least 6 visible fields per well or chamber slide containing HNECs. The data is expressed as the means and SEM of 5 independent experiments under static and flow conditions. Statistical analysis was performed by one-way ANOVA with Bonferroni's multiple comparison test (A and B). Fab_2_- fragments of an anti-human SREC-I antibody were also used to test the influence of SREC-I on bacterial adhesion under flow condition to CRNECs in ibidi chamber slide assays (C). 10 µg/ml of the SREC-I Fab_2_-fragments and isotype control were used in all assays. Attachment of the *S. aureus* wild-type (wt), and *tagO* mutant was evaluated microscopically by counting at least 6 visible fields per chamber slide containing CRNECs. The data represent mean and SEM of 5 independent experiments. Statistical analysis was performed by one-way ANOVA with Bonferroni's multiple comparison test. (C) We also tested the influence of SREC-I on the adhesion of other *S. aureus* strain backgrounds (D). Fab_2_- fragments of an anti-human SREC-I antibody were used under flow conditions in ibidi chamber slide assays with HNECS. 10 µg/ml of the SREC-I Fab_2_- fragments and isotype control were used in all assays. Attachment of the *S. aureus* Newman (wt), and SH1000 mutant was evaluated microscopically by counting at least 6 visible fields per chamber slide containing CRNECs. The data represent mean and SEM of 4 independent experiments. Statistical analysis was performed by one-way ANOVA with Bonferroni's multiple comparison test. (D) Significant differences between groups are indicated by one (*P*<0.05), two (*P*<0.01), or three (*P*<0.001) asterisks (*).

To study the role of WTA-SREC-I interaction in adhesion to epithelial cells, we performed microscopical assays with fluorescently labeled bacteria. We chose the *S. aureus* strain background Sa113 for most in vitro and in vivo assays because it colonized the nasal cavity of cotton rats (see below) significantly better than more virulent strains like e.g. USA300 or USA400 which also altered the integrity of epithelial monolayers in adhesion assays under static conditions (data not shown). WTA exerted an important role in the adhesion to HNECs as well as CRNECs, since especially under shear stress conditions a mutant lacking WTA (*tagO*) exhibited a strong defect in adhesion (Figure 3). This phenotype could be complemented by a wild-type copy of the *tagO* gene on a plasmid. With HNECs (Figure 3A) we detected a 54% reduced adhesion of *tagO* bacteria under static conditions when compared to wild-type bacteria at an MOI of 10. This phenotype could also be complemented. Under shear stress conditions the adhesion of the *tagO* mutant was reduced by 77%. Complementation of the *tagO* mutant nearly restored wild-type levels. With CRNECs we detected a 55% reduction of the *tagO* mutant adhesion when compared to wild-type *S. aureus* under static conditions (Figure 3B and Figure S6). Again, this reduction could be complemented by expressing a wild-type copy of the *tagO* gene in the *tagO* mutant. Under shear stress conditions adhesion of the *tagO* mutant was reduced by 67% when compared to the wild-type bacteria. Complementation of the *tagO* mutant did nearly restore wild type levels of adhesion (Figure 3B). With HNECs the adhesion of whole *S. aureus* bacterial cells could be partially blocked under static (Figure 4A) and shear stress condition (Figure 4B) using Fab_2_-fragments of a function blocking SREC-I antibody. Fab_2_-fragments of an isotype control had no impact on the adhesion of whole bacteria to HNECs. Under static conditions (Figure 4A), preincubation with the SREC-I Fab_2-_fragments decreased the adhesion of the wild-type by 67%. In contrast, incubation with the isotype control did not change the level of adhesion. The reduced level of *tagO* mutant adhesion was not further influenced by SREC-I antibody or isotype control preincubation. Under shear stress conditions (Figure 4B) preincubation with the SREC-I Fab_2-_fragments decreased adhesion of the wild-type to HNECs by 81%. Incubation with the isotype control did not significantly change the level of adhesion when compared to the tests without Fab_2_-fragments. The level of *tagO* mutant adhesion was not influenced by SREC-I antibody or isotype control preincubation. The SREC-I Fab_2_-fragment inhibited adhesion of whole *S. aureus* bacteria on CRNECs in a similar manner and adhesion decreased by 54% (Fig. 4c). Furthermore, the adhesion of additional *S. aureus* strains (Newman, SH1000, USA100, USA300 and USA400) could be blocked with the SREC-I FAb_2_-fragments, but not with isotype control Fab_2_-fragments (Fig. 4D).

**Figure 5:**
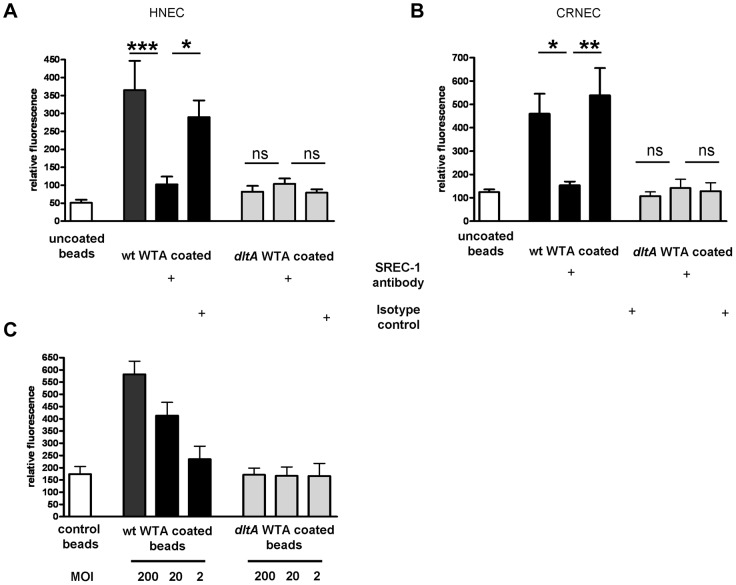
Impact of SREC-I on WTA-mediated adhesion to HNECs and CRNECs. The anti-human SREC-I antibody was used to test the influence of SREC-I on adhesion of WTA-coated latex beads under static condition in a 24 well microtiter plate assays on HNECs (A) and CRNECs (B). A ratio of 20 beads per epithelial cells was used in all depicted assays. 10 µg/ml of the anti-SREC-I antibody and isotype control were used to test the impact of SREC-I. Adhesion of the beads coated with wt WTA (HNECs and CRNECs) and *dltA* WTA (HNECs only) or control beads containing no WTA (HNECs and CRNECs) was measured in a fluoreader and expressed as relative fluorescence. The data are expressed as the means and SEM of 6 independent experiments. Statistical analysis was performed by one-way ANOVA with Bonferroni's multiple comparison test (A and B). The dose dependency of WTA-coated latex beads was tested in assays with CRNECs (C). Adhesion of the beads coated with wt WTA and *dltA* WTA or control beads containing no WTA was measured in a fluoreader and expressed as relative fluorescence. The data are expressed as the means and SEM of 5 independent experiments (C). Significant differences between groups are indicated by one (*P*<0.05), two (*P*<0.01), or three (*P*<0.001) asterisks (*).

Specific, WTA-mediated, adhesion to epithelial cells was demonstrated with WTA-coated fluorescent beads. Wild-type WTA-coated beads bound in a dose-dependent manner to HNECs as demonstrated before [18] and CRNECs (Fig. 5C) while beads coated with structurally altered *dltA* WTA (negatively charged) showed no specific adhesion to HNECs (Fig. 5A and [18]) and CRNECs (Fig. 5C). In line with bacterial adhesion assays, the function blocking SREC-I antibody revealed the impact of this receptor on WTA-dependent adhesion also in assays employing WTA-coated, fluorescently labeled latex beads (Figure 5A and B). The antibody blocked adhesion of WTA-coated beads to HNECs significantly while an isotype control had no impact on adhesion. In more detail, adhesion of wild-type WTA-coated beads to HNECs (Figure 5A), was reduced by 72% after SREC-I antibody preincubation while the isotype control had no influence on adhesion. Beads incubated with structurally altered *dltA* WTA lacking D-alanine side chain esters exhibited a decrease in adhesion by 78%. The SREC-I antibody and isotype control did not significantly influence adhesion of *dltA* WTA-coated beads. Control beads without WTA exhibited only a weak binding of 7.2% when compared to wild-type WTA-coated beads. With CRNECs the same SREC-I antibody blocked adhesion of WTA-coated beads in a similar manner (Figure 5B). Preincubation with the SREC-I antibody decreased adhesion by 67%. The isotype control had no significant impact on adhesion of WTA-coated beads to CRNECs (Figure 5B).

**Figure 6:**
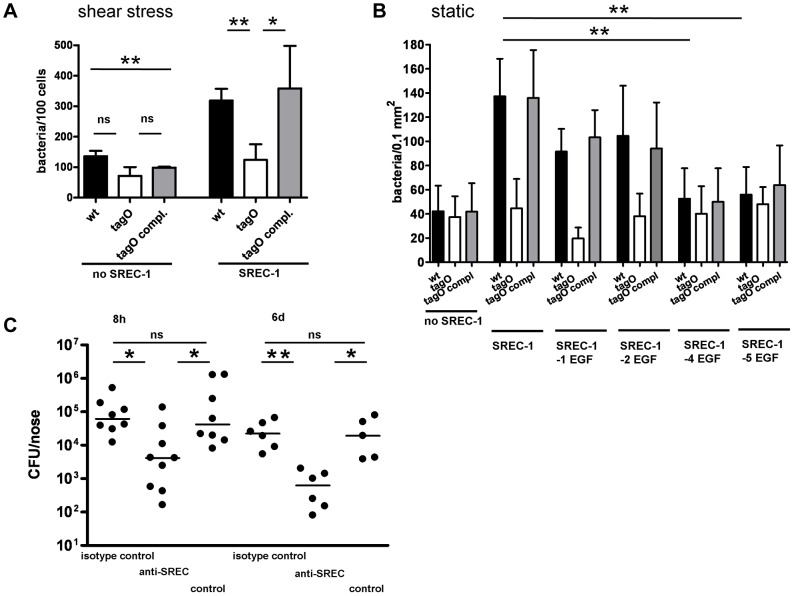
Impact of SREC-I on adhesion of *S. aureus* to CHO epithelial cells and impact of SREC-I on the early phases of nasal colonization in the cotton rat model. *S. aureus* adhesion to the cell line CHO expressing or not expressing SREC-I assayed in chamber slides under shear stress (A) and under static conditions (B). Attachment of *S. aureus* wt, *tagO*, and *tagO* compl. evaluated microscopically by counting at least 8 visible fields per well or chamber slide containing CHO cells without SREC-I or expressing full length SREC-I (A) or consecutive truncations of the extracellular EGF-like domains (B). Means and SD of 4 independent experiments (static) or 3 independent experiments (flow) are shown. Statistical analysis was performed by one-way ANOVA with Bonferroni's multiple comparison test (A and B). Significant differences are indicated by one (*P*<0.05), two (*P*<0.01), or three (*P*<0.001) asterisks (*). Nasal colonization was assayed in a cotton rat model (C). Bacterial numbers were determined 8 h and 6 days after inoculation. 15 min prior to inoculation cotton rats were pretreated with 2 µg anti-SREC-I Fab_2_-fragment per nose. After 8 h and 6 days the noses were dissected and the bacterial CFU was evaluated on *S. aureus* selective highchrome agar. Statistical analysis was performed by D'Agostino & Pearson omnibus normality test and a subsequent Kruskal-Wallis non-parametric ANOVA with Dunn's Multiple Comparison Test (C). Significant differences between groups are indicated by one (*P*<0.05), two (*P*<0.01), or three (*P*<0.001) asterisks (*).

Experiments with CHO epithelial cells expressing or not expressing functional SREC-I clearly underscore the role of WTA-SREC-I interaction in *S. aureus* adhesion to epithelial cells (Figure 6). Under flow conditions presence of SREC-I led to a strong increase in adhesion of wild-type *S. aureus* whereas no difference in the significantly lower adhesion of WTA-deficient *S. aureus* (*tagO* mutant) could be detected, irrespective of whether SREC-I was present or not (Figure 6A). Expression of full length SREC-I in CHO cells led to a statistically significant 2.3 fold increase in adhesion of wild-type bacteria. With SREC-I expressing CHO cells the *tagO* mutant exhibited a severely decreased adhesion (72% reduction) when compared to the wild-type. This residual adhesion was increased by a factor of 1.7 when SREC-I was expressed in the CHO cells although this increase wasn't statistically significant. The complemented mutant exhibited an adhesion profile that resembled the wild-type.

To localize the region of the SREC-I receptor-mediating interaction with WTA we used CHO cells expressing SREC-I with truncated EGF-like domains (Figure 6B). We assayed the adhesion of *S. aureus* wild-type, WTA-lacking *tagO* mutant and the complemented mutant under static condition. On CHO cells that express no SREC-I we detected 42±21, 37±17 and 42±24 bacteria/0.1 mm^2^ CHO cells for wild-type, *tagO* mutant and complemented mutant, respectively. Adhesion to CHO cells expressing SREC-I lacking the EGF domains until domain 4 was not altered when compared to full length SREC-I expressing cells. However, in CHO cells expressing a truncated SREC-I lacking the first 4 EGF-like domains we detected a significant drop in adhesion levels of 61% and 63% for wild-type and complemented mutant strain. The residual adhesion of the *tagO* mutant was not affected. These experiments, with CHO cells expressing successive truncations of SREC-I EGF-domains, led to the conclusion that the WTA interaction interface of SREC-I and WTA is located in the EGF domains 3 and 4 or between these domains (Figure 6B). This is in line with the presence of a stretch of charged amino acids (Figure S7) that could spatially alternate on both sides of a possible binding groove for WTA and therefore potentially represents the interaction surface of SREC-I and WTA. Interestingly preincubation of wild-type *S. aureus* with MgCl_2_ led to a decrease in adhesion to CRNECs which again argues for an impact of the WTA charge on SREC-I interaction (Figure S8A).

### SREC-I plays a key role in an in vivo model of nasal colonization

In order to demonstrate a possible impact of the WTA-SREC-I interaction on nasal colonization, we employed a cotton rat in vivo model. The cotton rat model of nasal colonization is a preferred, well established model for *S. aureus* since the nasal cavity of cotton rats shares histological properties with the human nasal cavity and permits long-term colonization experiments [29]. Most importantly, cotton rats, as opposed to mice, are susceptible to many human respiratory pathogens, and experimental diseases develop similar to those observed in humans [30]. We tested the impact of a function-blocking antibody and a corresponding isotype control in the early phases and late phases of nasal colonization (Figure 6C). At early time points (8 h) upon exposure to *S. aureus* the impact of adhesion factors that modulate primary adhesion should be obvious. We could detect a significant decrease in colonization after preincubation with Fab_2_-fragments of the SREC-I antibody but not with an isotype control. The decrease in *S. aureus* CFU after preincubation with the function-blocking antibody was comparable with the decrease in colonization observed before with a WTA-deficient *tagO* mutant [6], [18]. After 6 days, colonization in SREC-I antibody treated animals did not recover to the level observed in non treated animals or isotype control treated animals. The same effect could be observed in a different strain background. USA100 colonization of the cotton rat nares was significantly abrogated after pretreatment with Fab_2_ fragments of the SREC-I antibody (Figure S8B).

## Discussion

An integral step in the establishment and persistence of nasal colonization is the adhesion of bacteria to nasal epithelial cells and surface components of epithelial surfaces. Histological evidence led to the conclusion that *S. aureus* seems to predominantly colonize the anterior part of the nasal cavity (vestibulum nasi), which is lined by a stratified, keratinized, non-ciliated squamous epithelium [31], [32]. The upper cell layers in the anterior part comprise mostly anucleated, squamous cells termed corneocytes, which are highly keratinized and are surrounded by a proteinaceous structure containing loricrin and involucrin [33], [34]. However, these cellular layers are constantly shed, which should contribute to the clearing of attached bacteria. Therefore, interaction with the epithelial surface in the anterior part of the nasal cavity cannot fully explain persistent colonization. This idea is backed by newer evidence that demonstrates *S. aureus* interaction with living ciliated cells in deeper areas of the nasal cavity or even the throat [5], [6], [7], [8]. In addition bacterial numbers seem to be similar in all parts of the nasal cavity [9], [10]. In this line of thought, we investigated the molecular details of *S. aureus* adhesion to cells of the inner nasal cavity. Thus, we concentrated on primary growing cells isolated from the nasal cavity of humans and cotton rats. We detected expression of SREC-I, which is a member of the type F scavenger receptor family [25], in these cells. Our study is the first report of SREC-I expression in nasal epithelial cells. SREC-I exhibits six extracellular EGF-like domains, binds acetylated lipoproteins as a natural substrate [26], and has been identified as an important co-receptor for *Neisseria gonorrhoeae* adhesion to epithelial cells [27]. Our experiments revealed an impact of this receptor on *S. aureus* adhesion to nasal epithelial cells *in vitro* and *S. aureus* colonization in a cotton rat model of nasal colonization *in vivo*.

*S. aureus* nasal colonization is a multifactorial process and surface proteins of *S. aureus*, such as ClfB, show a considerable impact in animal models of nasal colonization [4], [14], [35], [36], [37]. In the cotton rat model of colonization these proteins seem to be more important for long-term colonization, whereas WTA impacts on the early stages while colonization is established [6]. This is in line with *ex vivo* analysis of the transcriptional level of WTA biosynthesis genes (*tagO* and *tarK*) and surface protein expressing genes [21], [22] which reveals a late stage upregulation of surface protein expressing genes.

Interestingly, WTA shows no influence on *S. aureus* interaction with keratinized cells in the anterior region of the nasal cavity but clearly governs part of the adhesion to growing epithelial cells isolated from deeper parts of the nasal cavity [6], [16].

We could show here that WTA specifically binds to SREC-I and that this facilitates adhesion of *S. aureus* to growing epithelial cells of the inner nasal cavity. In binding assays with immobilized WTA a functional SREC-I Fc-chimera, containing the extracellular domains of SREC-I fused to Fc-fragements, bound WTA in a dose dependent manner. This binding could be blocked by a SREC-I antibody. In adhesion assays with whole bacteria, *S. aureus* mutants that lacked WTA showed a significant defect, and the adhesion of wild-type *S. aureus* could be blocked with anti-SREC-I Fab_2_-fragments. The effect was especially pronounced under mild shear stress conditions. These conditions can be found in the nasal cavity where mucus movement, due to cillial activity and airstream, constantly creates shear stress conditions at the interface of bacteria and epithelial surfaces.

To understand the molecular details of SREC-I-WTA interaction we expressed truncated versions of SREC-I in CHO cells. Adhesion studies with these cells revealed a WTA binding interface on EGF-domains 3 and 4 of SREC-I. Wild-type WTA usually exhibits both positively and negatively charged residues in the ribitol-phosphate repeating units. Interestingly, experiments with negatively charged, *dltA* mutant derived WTA that lacks all D-alanine esters, indicate a role of the D-alanine modification in WTA/SREC-I interaction. Since we could only partially restore WTA-SREC-I interaction with the addition of Mg^2+^, WTA charge is important but can not be the only determinant of binding affinity. In line with this notion, inhibition experiments with purified WTA from wild-type and *dltA* mutant again demonstrate the role of the presence of D-alanine esters in the WTA structure. We therefore postulate that both the zwitterionic charge of the repeating units and the actual spacing of the charges are required for SREC-I binding. Charged amino acid residues in the WTA binding interface of SREC-I are most likely responsible for this interaction. Mutational studies of SREC-I are part of the ongoing research in our laboratory and will reveal the structural requirements for the WTA-SREC-I interaction.

WTA is one of the most abundant molecules in the staphylococcal cell wall and therefore might facilitate multiple interactions of lower affinity which could create kinetically more favourable conditions for *S. aureus* binding proteins. Thus, we postulate that WTA modulates the strength of an initial interaction between bacterium and an epithelial surface, thereby facilitating binding protein activity.

**Figure 7:**
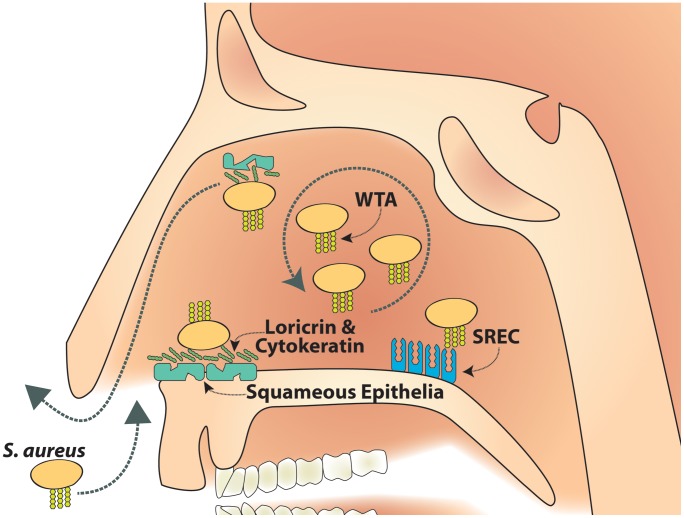
Model of SREC-I role in nasal colonization. SREC-I can be detected on growing nasal epithelial cells in the inner nasal cavity. *S. aureus* WTA binds to SREC-I which mediates *S. aureus* adhesion to cells from the inner nasal cavity. This WTA/SREC-I interaction has a considerable impact on nasal colonization. Therefore, we postulate a reservoir of *S. aureus* cells in the inner nasal cavity as a source for continuous colonization, which is at least partially maintained by WTA dependent adhesion to epithelial cells.

In the cotton rat model the function blocking anti-SREC-I antibody had a significant impact on the initial colonization of the nasal cavity with *S. aureus* wild-type bacteria. Therefore, it is tempting to conclude that in the early stages of colonization, the initial interaction of *S. aureus* with nasal surfaces partially takes place at other sides than the very anterior part of the nasal cavity. In addition, during later stages and persistent colonization, the inner part of the nasal cavity likely represents one of several habitates in the nose [9]. Keeping in mind that cells of the very anterior epithelial linings are shed and permanently removed from the nasal cavity, bacteria that enter deeper zones of the nasal cavity might have better chances to overcome physiological barriers, proliferate and then disseminate again into other areas of the nasal cavity, including the anterior (Figure 7). In line with this idea, histological evidence and *in vitro* studies suggest that *S. aureus* can also bind to nasal epithelial cells deeper inside the nasal cavity [6], [38] and is also taken up by and able to persist within these cells *in vivo* [5].

Taken together we identified SREC-I as a receptor for WTA on nasal epithelial cells of human and cotton rat origin. SREC-I binds WTA most likely through charge interactions. This WTA-SREC-I interaction plays a key role in the initial stages of nasal colonization in a cotton rat model. This first report of a bacterial surface polymer-type-F scavenger receptor interaction could spark a new interest in charged bacterial surface polymers and their impact on bacterial interaction with the host. In the particular case of *S. aureus* it is worthwhile to speculate that the WTA-SREC-I interaction might be an interesting target for substances, like the antibody we used in this study, which could be used to combat *S. aureus* nasal colonization. At least it should be possible to use such substances as topical treatments, to prevent re-colonization after eradication of *S. aureus* in risk patients that require extended hospitalization. Such an approach could be also used in combination with vaccination therapy that targets other factors involved in *S. aureus* nasal colonization [37]. This could significantly reduce invasive *S. aureus* infections during hospitalization. In addition, further studies are now required to understand the impact of SREC-I on nasal colonization from an epidemiological perspective. We are currently trying to identify putative functional polymorphisms in SREC-I and correlate these with nasal carrier status. However, since nasal colonization is a multifactorial process, polymorphisms in SREC-I will be only one possible genetic determinant that has to be investigated in conjunction with other modulators of nasal colonization.

## Materials and Methods

### Bacterial strains

*S. aureus* SA113 (ATCC 35556) is a frequently used laboratory strain [39]. The mutant Δ*tagO* [18] was generated in this strain background by replacing the *tagO* gene with an erythromycin resistance cassette. This mutant is devoid of WTA [18]. Plasmid pRB*tagO* contains a wild-type copy of *tagO* and its promoter, and reconstitutes wild-type level WTA biosynthesis along with all relevant phenotypic properties [18]. An isogenic *S. aureus* Δ*dlt*A mutant [40] was used as a control in the affinity blotting experiments. Strains Newman (NCTC 8178) [41] and SH1000 [42] are frequently used laboratory strains. We also included strains USA100 [43], USA300 [44] and USA400 [44] in selected experiments.

### Recombinant DNA constructs

The GFP-tagged version of SREC-I was constructed by amplifying the cDNA-clone of wt-SREC-I (IRATp970A0758D6, RZPD: Deutsches Ressourcenzentrum für Genforschung GmbH) with primers SREC-I sense 5′-GACTAGATCT GACGAACCCG AGCGCTGCCA CCG-3′ and SREC-I antisense 5′-GACTGAATTC GTTCTGTTGG CCTGGAGATGG-3′. The resulting PCR fragments were cloned into pGEM-T easy TA-vector system (Promega) and transferred by BglII/EcoRI digestion and ligation into pEGFP-N1 (Clontech) resulting in GFP fused to the carboxy-terminus of the expressed proteins. Truncated SREC-I constructs were generated as follows: the amino-terminal domain including the signal peptide was amplified with SREC-I sense primer and SREC-SP-antisense (5′-GACTCTCGAG TTGATCCTTC TGCCTCCAGC CTG-3′). The C-terminal domain was sequentially truncated by PCR amplification using SREC-I antisense primer together with the different sense primers SREC-I-1EGF (5′-GACTCTCGAG TCCGCTGCCC GGCCCAGTAC TG-3′), SREC-I-2EGF (5′-GACTCTCGAG TTCCCGTGCG CCTGCGGCCC CC-3′), SREC-I-4EGF (5′-GACTCTCGAG CTGCCCTGCC CGGCAGGCAG CC-3′) or SREC-I-5EGF (5′-GACTCTCGAG GGACCCCTGC CCCACTGGTA CC-3′), respectively. The resulting PCR fragments were cloned into pGEM-T easy TA-vector system. DNA fragments containing either the signal peptide or truncated versions of SREC-I were prepared by restriction with BglII/XhoI or XhoI/EcoRI, respectively and were ligated into pEGFP-N1. CHO cells were transfected with the resulting constructs as well as with the empty vector pEGFP-N1. Stable cell clones were isolated and maintained in Ham's F12 medium (Gibco) supplemented with 10% FCS and 400 µg/ml G418.

### Labeling of human SREC-I

Recombinant SREC-I Fc-chimera was purchased from R&D-Systems. The molecule was reconstituted in PBS to a concentration of 100 µg/ml. EZ-link Sulfo-NHS-LC-Biotin (Pierce) was used at a concentration of 50 µg/ml. The biotinylation reaction was performed in a modified PBS-buffer (pH = 8.5) for 30 min at 37°C. The reaction was stopped by extensively dialyzing against PBS at a pH = 7 in a Slide-A-Lyzer dialysis cassette (Pierce).

In addition SREC-I was labelled with FITC. Therefore 100 µl of a FITC-solution (40 µM) in carbonate-buffer (pH = 8.5) was mixed with a SREC-I at a concentration of 100 µg/ml and allowed to react for 1 h at 37°C. After that time period the reaction was stopped by dialysis against PBS in a Slide-A-Lyzer dialysis cassette (Pierce). We also labeled human recombinant CD36 (R&D systems) with the same protocol.

### Cell lines and primary cells

Primary human nasal epithelial cells (HNECs) were purchased from Provitro (Berlin) and cultivated in airway epithelial growth medium (Provitro). HNECs were used up to passage 6. Primary cotton rat nasal epithelial cells (CRNECs) were isolated from cotton rat noses. Cotton rats noses were digested ON in DMEM with 10% FCS (Sigma), penicillin (100 U/ml), streptomycin (100 µg/ml; Gibco,), and Collagenase Type VIII (Sigma). Then tissue was transferred in DMEM culture medium containing 10% FCS, penicillin (100 U/ml), and streptomycin (100 µg/ml). After a significant amount of cells were detectable (72 h) cells were detached with Trypsin (Sigma) and subcultured in DMEM supplemented with 10% FCS (Sigma). Growth rate and morphology was constantly monitored and cells were used up to passage 7.

### Detection of SREC-I on HNEC and CRNEC cells by FACS-analysis and immunofluorescence

Studies were performed as described before [45]. Cells were detached by scraping in PBS supplemented with 1% FCS. SREC-I was detected by using an anti human SREC mouse IgG2B antibody (R&D). Cells were blocked with 10% FCS in PBS for 20 min at room temperature following incubation with the anti-SREC antibody for 45 min at 4°C. Subsequently, cells were washed twice and incubated for 45 min at 4°C with secondary antibody (anti-mouse IgG –PE). Flow cytometric analysis was performed using a BD FacsCalibur. As an isotype control we used a mouse IgG2B (R&D systems).

For immunofluorescence detection of SREC-1 on HNECS and CRNECs, cells were cultivated in *Lab-TekII-Slides* (Thermo) and were fixed with 100% MeOH pa for 10 min at -20°C. 1% BSA (in PBS) was added to the cells to block nonspecific staining. The cells were incubated at room temperature for 1 h. Then the cells were incubated with 10 µg/ml SREC-1 antibody (SREC-I/SR-F1 goat IgG polyclonal antibody; R&D), 10 µg/ml isotype control (Normal goat IgG; R&D systems), or blocking buffer (1% BSA in PBS) for 1 h at room temperature and washed three times with PBS. A donkey anti-Goat IgG-Cy5 (Milipore) was used as secondary antibody (diluted 1∶75). Nuclei were stained with 1 µg/ml DAPI (Applicam) and cytoskeleton was stained with phalloidin-TRITC (SIGMA) for 5 min at room temperature. After three washing steps with PBS the cells were mounted with Mowiol. Microscopy was performed on a Zeiss LSM-710 NLO with Zen 2011 software.

### Detection of SREC-I mRNA by RT-PCR

The SREC gene in CRNECs and HNECs was detected via RT-PCR. RNA was isolated with the RNeasy Mini Kit (Qiagen). To exclude DNA contamination, RNA was digested with 10 U of RNase-free DNase I (Roche) and 40 U of RNasin ribonuclease inhibitor (Promega) for 30 min at room temperature. DNase I treatment was stopped using DNase Inactivation reagent (Ambion). RNA was transcribed using First Strand cDNA Synthesis Kit (Fermantas) and 200 ng of random hexamer primers. PCR for detection of the SREC gene this cDNA was amplified with following primers. The sequence primers designed using the mouse SREC-I sequence were SREC-m1 fwd 5′AGCTGCCTTGCAACCCTGGA and SREC-m1 rev5′AGGTGCCTGCAGGACAT GGC, respectively. The second primer pair were SREC-m2 fwd 5′TGGGACTAGAGCTGGTGTTCT and SREC-m2 re5′CAGATGGGGATGGTGCA TTCT. The primers designed based on the human SREC-I sequence were SREC-h1 fwd 5′TGAAGCCGGGCCTCTGTCGA and SREC-h1 rev5′ CAGATGGGGATGGTG CATTCT as well as SREC-h2 fwd 5′CC TGCCAGAAAGACGAGGTG and SREC-h2 rev5′CCAGGCTTGCATCGACAGAG.

### Binding blots

Purified WTA was blotted on a nitrocellulose membrane by pipetting. 5 µl of a WTA solution in 20 mM NaAcetat containing 200, 100, 50, 25 and 12.5 nmol Pi were spotted onto the membrane. The blots were blocked in TBS containing 5% BSA. After 3 washes with TBS-0.1% Tween20 the blots were incubated with 500 mg/ml SREC-I Fc-chimera (R&D systems) in TBS at room temperature for 30 min. After 1 wash with TBS-0.1% Tween20 the blots were incubated with an anti-human IgG antibody coupled with a 800 nm emitting infrared dye (LI-COR) for 45 min. The blots were washed again once with TSB-0.1% Tween20 and once with PBS and SREC/WTA interaction was detected on a LI-COR Odyssey system.

### Binding studies of soluble SREC-I to bacteria

Bacteria were cultured over night in MHB media and subcultured until mid-logarithmic growth phase in IMDM. The Bacteria were washed 3 times with PBS. The assay was performed in a 300 µl scale and 1*10^8^ bacteria were incubated with 50 µg/ml FITC labeled SREC-I Fc-chimera (R&D systems) in PBS. After 30 min at 37°C and slow shaking, the bacteria were harvested by centrifugation at 10000 rpm for 10 minutes. The bound fluorescence was calculated by subtracting the unbound fluorescence from the total fluorescence of the FITC labeled SREC-I solution. In some assays different MgCl_2_ concentrations or purified WTA were added to test the charge dependency of the WTA/SREC-I interaction. As a specificity control FITC labeled human CD36 (R&D systems) was used at 50 µg/ml.

### Adherence of WTA-coated beads or whole bacteria to CRNECs and HNECs

CRNECs were seeded in 24 well culture plates at 2.5–5×10^4^ cells/well in Dulbecco's Modified Eagle Medium (DMEM; PAA Laboratories) with 10% FCS, 100 U/ml penicillin, and 100 µg/ml streptomycin, while HNECs (Provitro) were seeded at 5×10^4^ cells/well in airway epithelial growth medium (Provitro). The Plates were incubated at 37°C under 5% CO_2_. Binding assays where performed as described before [18]. When blocking experiments were performed, 10 µg/ml anti-human SREC-I/SR-F1 antibody (R&D systems) or 10 µg/ml isotype control (normal goat IgG; R&D systems) were used in adhesion assays with WTA coated latex beads (WTA passively adsorbed to amine modified latex beads (Sigma) as described before [18]). The plates were incubated for 30 min at 37°C. The antibody concentrations were evaluated by dose dependency experiments (data not shown). WTA-coated latex beads (wild type WTA and negatively charged *dltA* WTA) and the control sample (only beads) were diluted in RPMI 1640. The fluorescence of the WTA-coated beads and the control sample was adjusted accordingly. Then, the samples were used in adhesion assays on epithelial cells with bead/epithelial cell ratios of 200∶1, 20∶1, 2∶1 for dose dependency experiments. In antibody blocking assays a ratio of 20∶1 was used. The plates were incubated for 1 h at 37°C. After a washing step with PBS the relative fluorescence at 520 nm (emission) was quantified using a fluororeader (BMG Labtech). At least five assays were run in triplicate. When bacteria where used, bacteria were prepared and labeled with fluorescein isothiocyanate (FITC) as described before [18]. For Cell numbers were adjusted using a Neubauer chamber. 5 independent assays were performed under static conditions. When blocking experiments were performed, 10 µg/ml Fab_2_-fragments of anti-human SREC-I/SR-F1 antibody (R&D systems) or 10 µg/ml isotype control (normal goat IgG; R&D systems) were used. Fab_2_-fragments were produced with a Pierce kit according to the manufacturer's description. When assays under flow conditions were performed epithelial cells were seeded in chamber slides (μ-Slide VI0.4; Ibidi). For infection peristaltic pumps (Amersham) were used with flow conditions mimicking 0.5 dynes, according to the manufacturer's instructions with a MOI of 20 (at 37°C under 5% CO_2_) for 30 min. 6 independent assays were performed under flow conditions

### Adhesion assays on CHO epithelial cells expressing full length, truncated, or no SREC1-receptor under static and flow conditions in chamber slides

Chinese hamster ovary (CHO) epithelial cells were seeded in chamber slides (μ-Slide VI0.4; Ibidi) at 1×10^5^ CHO cells/well and in DMEM/F12-GlutaMaxTM-I medium (GIBCO) supplemented with 10% fetal calf serum (FCS), 400 µg/ml geneticin (G418), 100 U/ml penicillin, and 100 µg/ml streptomycin and grown at 37°C under 5% CO_2_. Bacteria were prepared and labeled with fluorescein isothiocyanate (FITC) as decribed before [18]. Adhesion to CHO cells containing the empty vector pEGFP-N1, the vector with the full length SREC-I receptor, or the vector with a truncated SREC-I receptor was assayed with FITC labeled *Staphylococcus aureus* SA113 wild-type, *S. aureus* SA113 Δ*tagO*, or *S. aureus* SA113 Δ*tagO* pRB*tagO* at a MOI of 10. After the infected cells were incubated for 1 hour at 37°C under 5% CO_2_ the cells were washed three times with phosphate-buffered saline (PBS) and then fixed with 4% paraformaldehyde (PFA). Adherent bacteria/0.1 mm^2^ were counted using a fluorescence microscope (Zeiss). Three assays were run in duplicate.

Before assays under flow conditions were performed epithelial cells were seeded in chamber slides (μ-Slide VI0.4; Ibidi) at 6×10^4^ CHO cells/well and cultivated as described above. For infection peristaltic pumps were used with flow conditions mimicking 0.5 dynes according to the manufacturer's instructions with a MOI of 20 (at 37°C under 5% CO_2_) for 30 min. Adherent bacteria/100 epithelial cells were counted using a fluorescence microscope. Three independent assays were performed.

### Cotton rat model of nasal colonization

The cotton rat model was used as described earlier [18]. Cotton rats were anesthetized and noses were preincubated for 15 min with 2 µg of anti-human SREC-I Fab_2_-fragment (R&D) per nose. Afterwards, the noses were instilled intranasally with 10 µl of 1×10^9^ colony forming units (CFU) of *S. aureus* (SA113). 8 hours after bacterial instillation the animals were euthanized and noses were removed surgically. The noses were vortexed in 1 ml of PBS containing 0.5% Tween for 30 sec. Samples were plated on appropriate agar plates and the bacterial CFU was determined. All animals received drinking water with 2.5 mg/ml streptomycin continuously, starting 3 days prior before beginning the experiment to reduce the natural nasal flora.

### Ethics statement

Animal experiments were performed in strict accordance with the German regulations of the Society for Laboratory Animal Science *(GV-SOLAS)* and the European Health Law of the Federation of Laboratory Animal Science Associations (FELASA). The protocol was approved by the Regierungspräsidium Tübingen (Permit Numbers: T1/10)).

### Statistical analysis

Statistical analyses were performed with Graphpad Prism, using appropriate statistical methods as indicated. *P* values≤0.05 were considered significant.

## Supporting Information

Figure S1 **WTA structure and biosynthesis.** WTA is a cell wall glycopolymer with a negatively charged ribitol-phosphate backbone. The ribitol units are modified with positively charged D-alanine esters (renders the polymer zwitterionic) and GlcNAc residues in α or β-configuration. WTA biosynthesis occurs directly at the cytoplasmic membrane, starting with the addition of Glc*N*Ac-P from UDP-Glc*N*Ac to undecaprenol-phosphate by the TagO enzyme. A *tagO* mutant lacks all WTA in the cell wall. After the addition of Man*N*Ac, the anchor structure is finished by adding 3 glycerol-phosphate molecules. Then up to 40 ribitol-phosphate molecules are polymerized step-wise until the WTA molecule is finished and finally transported across the membrane by TagGH. The mature polymer is linked to the C6 atom of MurNAc in the the peptidoglycan and then modified with GlcNAc and D-alanine. The addition of D-alaline esters are performed by the gene products of the *dlt*-operon. A *dltA* mutants lacks the D-alanylation and therefore exhibits negatively charged WTA.
(TIF)

Figure S2 **Expression of SREC-I in HNECs and CRNECs.** Primary cotton rat nasal epithelial cells (CRNECs) were isolated from nasal turbinates and monitored under culture condition for morphological integrity by microscopy (A). Surface exposure of SREC-I on HNECs and CRNECs was monitored by FACS with the same anti-human SREC-I antibody and appropriate isotype control. A PE labelled anti-mouse IgG antibody was used as the secondary antibody. A representative experiment is shown here (B) Expression of SREC-I in HNECs and CRNECs was assayed with RT-PCR. Primer sets derived from the human and murine SREC-I sequence were used (C). The house keeping gene GAPDH was used as a control.
(TIF)

Figure S3 **Surface presentation of SREC-I on CRNECs and HNECs monitored by confocal microscopy.** The appropriate isotype control (A) and a mouse derived anti-human SREC-I antibody (B) were used to stain SREC-I on the cellular surface (upper panel CRNECs and lower panel HNECs respectively). Cell nuclei were stained with DAPI. Bars represent 20 µm.
(TIF)

Figure S4 **Direct binding of SREC-I to WTA.** WTA was spotted in increasing concentrations on a nitrocellulose membrane (wild-type WTA squares, *dltA* WTA triangles) and interaction with a SREC-I Fc-chimera (500 ng/ml) was measured. Bound SREC-I Fc-chimera was detected with a secondary antibody coupled to an 800 nm emitting infrared dye on a LI-COR Odyssey infrared imaging system. The mean and SD of 3 independent experiments are shown.
(TIF)

Figure S5 **Charge dependency and specificity of SREC-I binding to WTA.** Binding of SREC-I to whole bacterial cells, measured with a FITC-labeled SREC-I Fc-chimera (A). Wild-type *S. aureus* and a *dltA* mutant with negatively charged WTA were incubated with different concentrations of MgCl_2_ and 50 µg/ml FITC-labeled SREC-I. In addition we added WTA purified from wild-type *S. aureus* and dltA mutant. The bound fluorescence was calculated by subtracting the unbound fluorescence from the total fluorescence of the FITC-labeled SREC-I solution. The mean and SD of 4 independent experiments are shown. Statistical analysis was performed by one-way ANOVA with Bonferroni's multiple comparison test (A). Comparison of SREC-I WTA specific binding with CD36 binding to whole bacterial cells. (B). Wild-type *S. aureus, tagO* mutant (no WTA) and *dltA* mutant with negatively charged WTA were incubated with 50 µg/ml of FITC-labeled SREC-I or FITC labeled CD36. The bound fluorescence was calculated by subtracting the unbound fluorescence from the total fluorescence of the FITC-labeled SREC-I solution. The mean and SD of 6 independent experiments are shown. Statistical analysis was performed by one-way ANOVA with Bonferroni's multiple comparison test. Significant differences vs. wild-type without MgCl_2_ are indicated by one (*P*<0.05), two (*P*<0.01), or three (*P*<0.001) asterisks (*).
(TIF)

Figure S6 **Confocal microscopy of** ***S. aureus*** **adhesion to CRNECs.** Green fluorescent *S. aureus* (FITC labeled) wild-type (A) and *tagO* mutant (B) cells where allowed to adhere to cotton rat nasal epithelial cells (CRNECs) under mild shear stress condition (0.5 dynes) in ibidi-chamber slides. Cells were stained with DAPI and phalloidin-TRITC. Bars represent 20 µm.
(TIF)

Figure S7 **Amino acid sequence of SREC-I with putative WTA binding site.** Negatively charged amino acids are labeled in red, positively charged amino acids in yellow (A). Model of WTA-SREC-I interaction. Both the zwitterionic charge of the WTA repeating units and the actual spacing of the charges are required for SREC-I binding (B).
(TIF)

Figure S8 **Charge dependency of WTA dependent adhesion to nasal epithelial cells and SREC-I dependent modulation of USA100 nasal colonization.** Adhesion to CRNECs, under shear stress conditions in ibidi chamber slide assays, was monitored after preincubation of the bacterial inoculums with different MgCl_2_ concentrations. The mean and SD of 4 independent experiments are shown. Statistical analysis was performed by one-way ANOVA with Bonferroni's multiple comparison test (A). Nasal colonization in the cotton rat model was tested with USA100 (B). Bacterial numbers were determined 6 days after inoculation. 15 min prior to inoculation cotton rats were pretreated with 2 µg anti-SREC-I Fab_2_-fragment per nose. After 6 days the noses were dissected and the bacterial CFU was evaluated on *S. aureus* selective highchrome agar. Statistical analysis was performed by one-way ANOVA with Bonferroni's multiple comparison test (B). Significant differences vs. wild-type without MgCl_2_ are indicated by one (*P*<0.05), two (*P*<0.01), or three (*P*<0.001) asterisks (*).
(TIF)
