# Correction: A Nasal Epithelial Receptor for *Staphylococcus aureus* WTA Governs Adhesion to Epithelial Cells and Modulates Nasal Colonization

**DOI:** 10.1371/journal.ppat.1004247

**Published:** 2014-06-13

**Authors:** The PLOS Pathogens Staff

The third author's name is spelled incorrectly. The correct name is: Michaela Faulstich. The citation from the original article remains the same.
