# Supplementary figures and images for: A Nasal Epithelial Receptor for Staphylococcus aureus WTA Governs Adhesion to Epithelial Cells and Modulates Nasal Colonization
Source: PLoS Pathog. 2014 May 1;10(5):e1004089. doi: 10.1371/journal.ppat.1004089 (PMC4006915; doi:10.1371/journal.ppat.1004089)

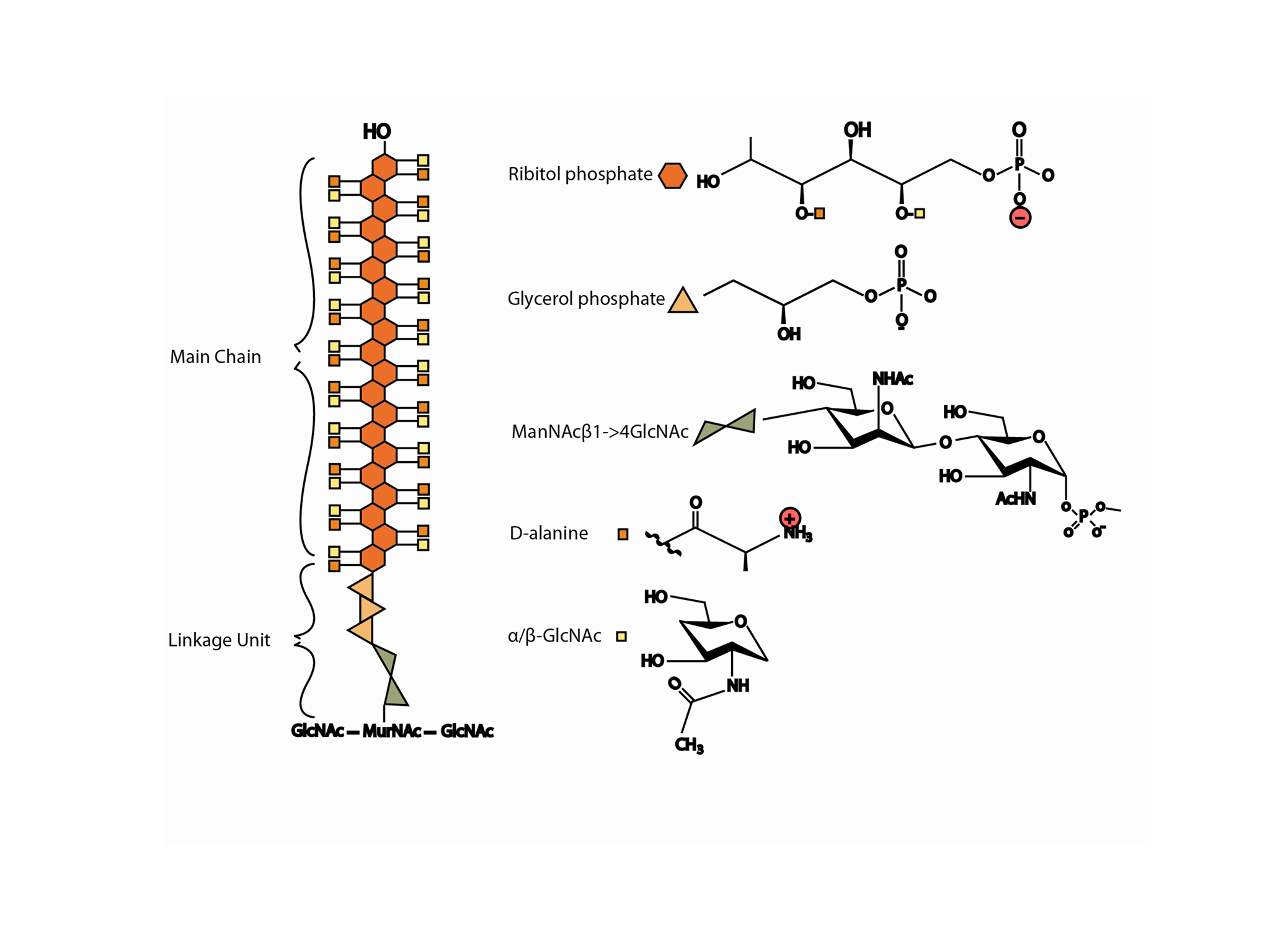

Supplement: Figure S1 — WTA structure and biosynthesis. WTA is a cell wall glycopolymer with a negatively charged ribitol-phosphate backbone. The ribitol units are modified with positively charged D-alanine esters (renders the polymer zwitterionic) and GlcNAc residues in α or β-configuration. WTA biosynthesis occurs directly at the cytoplasmic membrane, starting with the addition of GlcNAc-P from UDP-GlcNAc to undecaprenol-phosphate by the TagO enzyme. A tagO mutant lacks all WTA in the cell wall. After the addition of ManNAc, the anchor structure is finished by adding 3 glycerol-phosphate molecules. Then up to 40 ribitol-phosphate molecules are polymerized step-wise until the WTA molecule is finished and finally transported across the membrane by TagGH. The mature polymer is linked to the C6 atom of MurNAc in the the peptidoglycan and then modified with GlcNAc and D-alanine. The addition of D-alaline esters are performed by the gene products of the dlt-operon. A dltA mutants lacks the D-alanylation and therefore exhibits negatively charged WTA. (TIF) [file ppat.1004089.s001.tif]

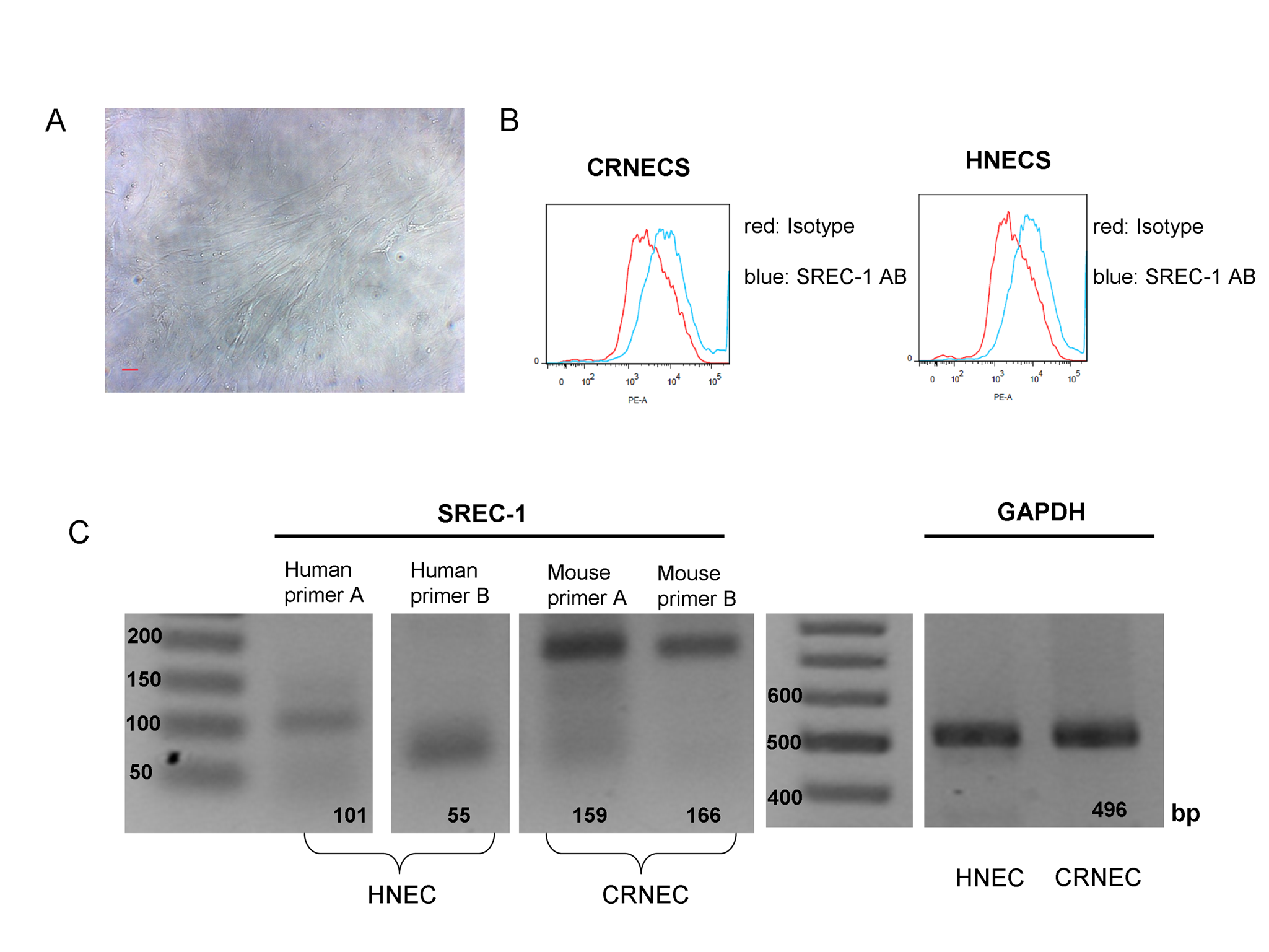

Supplement: Figure S2 — Expression of SREC-I in HNECs and CRNECs. Primary cotton rat nasal epithelial cells (CRNECs) were isolated from nasal turbinates and monitored under culture condition for morphological integrity by microscopy (A). Surface exposure of SREC-I on HNECs and CRNECs was monitored by FACS with the same anti-human SREC-I antibody and appropriate isotype control. A PE labelled anti-mouse IgG antibody was used as the secondary antibody. A representative experiment is shown here (B) Expression of SREC-I in HNECs and CRNECs was assayed with RT-PCR. Primer sets derived from the human and murine SREC-I sequence were used (C). The house keeping gene GAPDH was used as a control. (TIF) [file ppat.1004089.s002.tif]

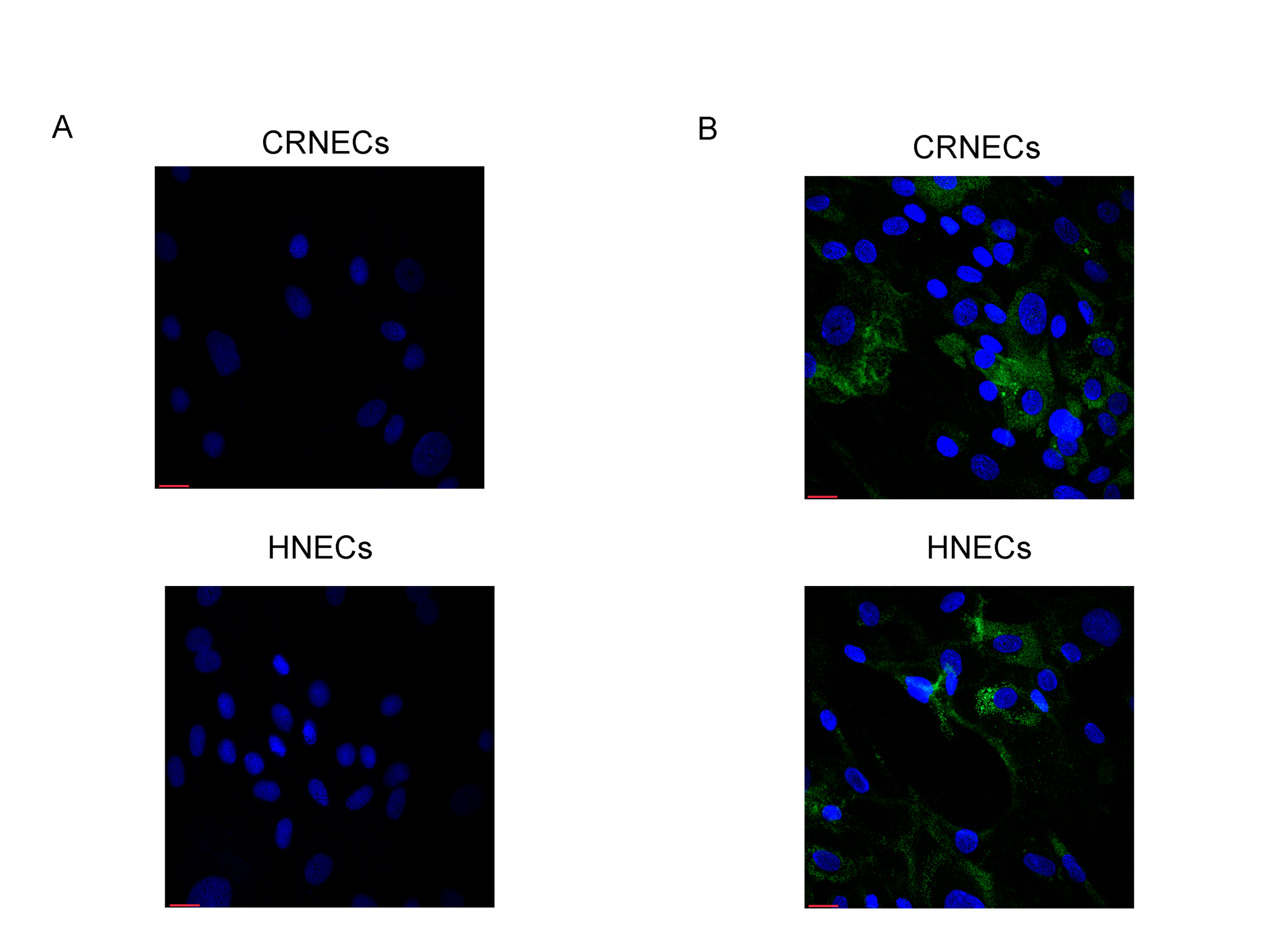

Supplement: Figure S3 — Surface presentation of SREC-I on CRNECs and HNECs monitored by confocal microscopy. The appropriate isotype control (A) and a mouse derived anti-human SREC-I antibody (B) were used to stain SREC-I on the cellular surface (upper panel CRNECs and lower panel HNECs respectively). Cell nuclei were stained with DAPI. Bars represent 20 µm. (TIF) [file ppat.1004089.s003.tif]

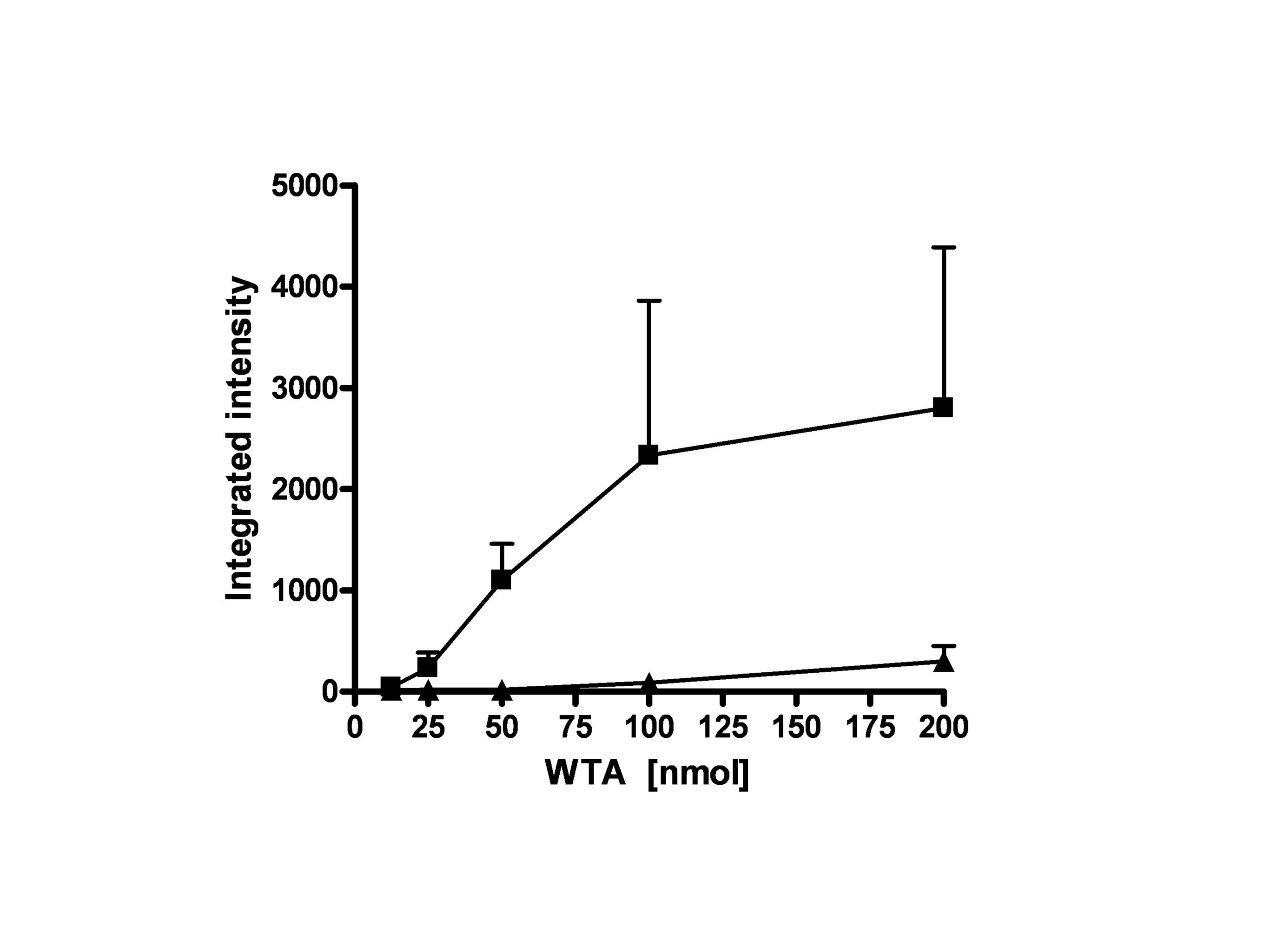

Supplement: Figure S4 — Direct binding of SREC-I to WTA. WTA was spotted in increasing concentrations on a nitrocellulose membrane (wild-type WTA squares, dltA WTA triangles) and interaction with a SREC-I Fc-chimera (500 ng/ml) was measured. Bound SREC-I Fc-chimera was detected with a secondary antibody coupled to an 800 nm emitting infrared dye on a LI-COR Odyssey infrared imaging system. The mean and SD of 3 independent experiments are shown. (TIF) [file ppat.1004089.s004.tif]

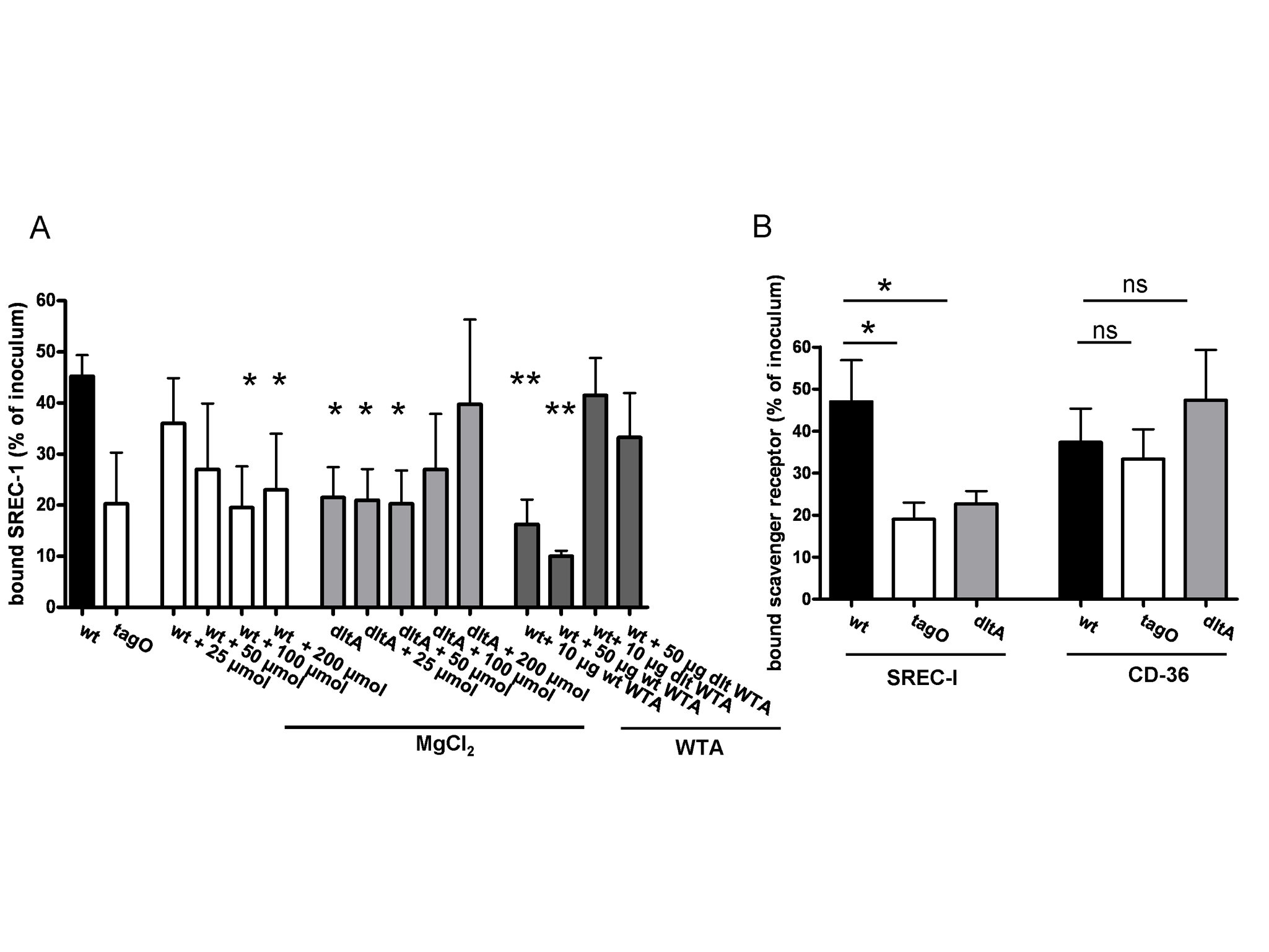

Supplement: Figure S5 — Charge dependency and specificity of SREC-I binding to WTA. Binding of SREC-I to whole bacterial cells, measured with a FITC-labeled SREC-I Fc-chimera (A). Wild-type S. aureus and a dltA mutant with negatively charged WTA were incubated with different concentrations of MgCl2 and 50 µg/ml FITC-labeled SREC-I. In addition we added WTA purified from wild-type S. aureus and dltA mutant. The bound fluorescence was calculated by subtracting the unbound fluorescence from the total fluorescence of the FITC-labeled SREC-I solution. The mean and SD of 4 independent experiments are shown. Statistical analysis was performed by one-way ANOVA with Bonferroni's multiple comparison test (A). Comparison of SREC-I WTA specific binding with CD36 binding to whole bacterial cells. (B). Wild-type S. aureus, tagO mutant (no WTA) and dltA mutant with negatively charged WTA were incubated with 50 µg/ml of FITC-labeled SREC-I or FITC labeled CD36. The bound fluorescence was calculated by subtracting the unbound fluorescence from the total fluorescence of the FITC-labeled SREC-I solution. The mean and SD of 6 independent experiments are shown. Statistical analysis was performed by one-way ANOVA with Bonferroni's multiple comparison test. Significant differences vs. wild-type without MgCl2 are indicated by one (P<0.05), two (P<0.01), or three (P<0.001) asterisks (*). (TIF) [file ppat.1004089.s005.tif]

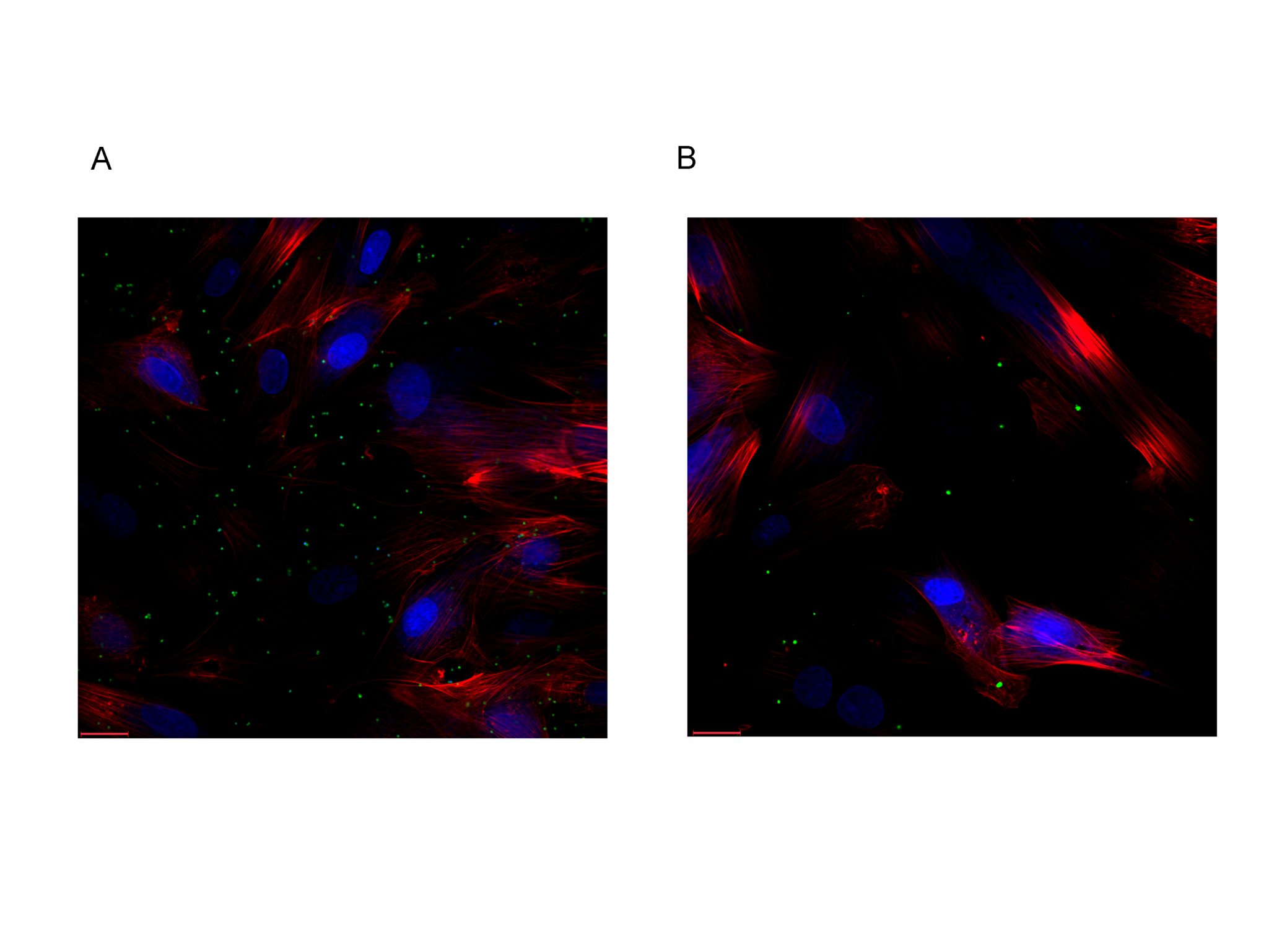

Supplement: Figure S6 — Confocal microscopy of S. aureus adhesion to CRNECs. Green fluorescent S. aureus (FITC labeled) wild-type (A) and tagO mutant (B) cells where allowed to adhere to cotton rat nasal epithelial cells (CRNECs) under mild shear stress condition (0.5 dynes) in ibidi-chamber slides. Cells were stained with DAPI and phalloidin-TRITC. Bars represent 20 µm. (TIF) [file ppat.1004089.s006.tif]

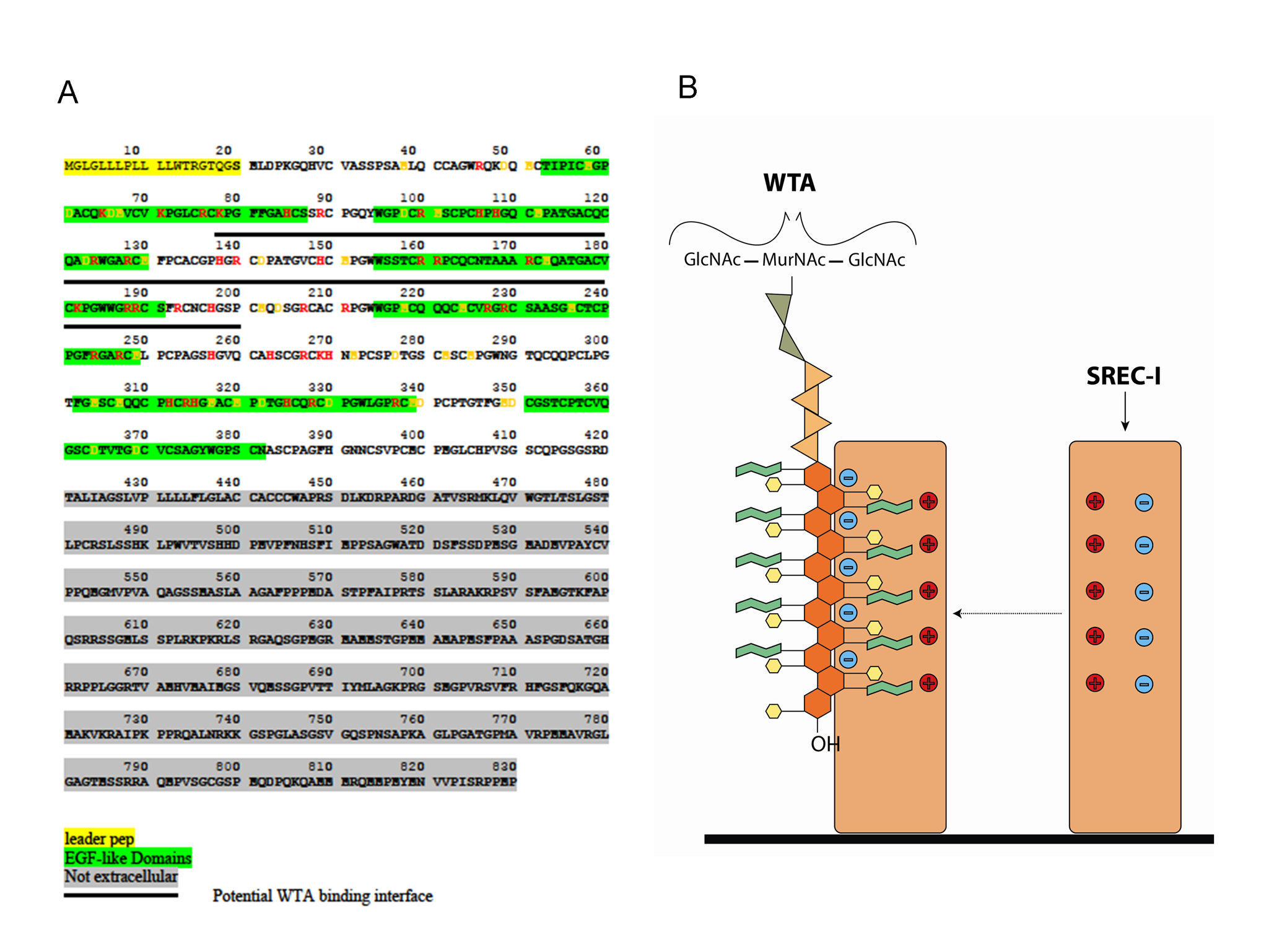

Supplement: Figure S7 — Amino acid sequence of SREC-I with putative WTA binding site. Negatively charged amino acids are labeled in red, positively charged amino acids in yellow (A). Model of WTA-SREC-I interaction. Both the zwitterionic charge of the WTA repeating units and the actual spacing of the charges are required for SREC-I binding (B). (TIF) [file ppat.1004089.s007.tif]

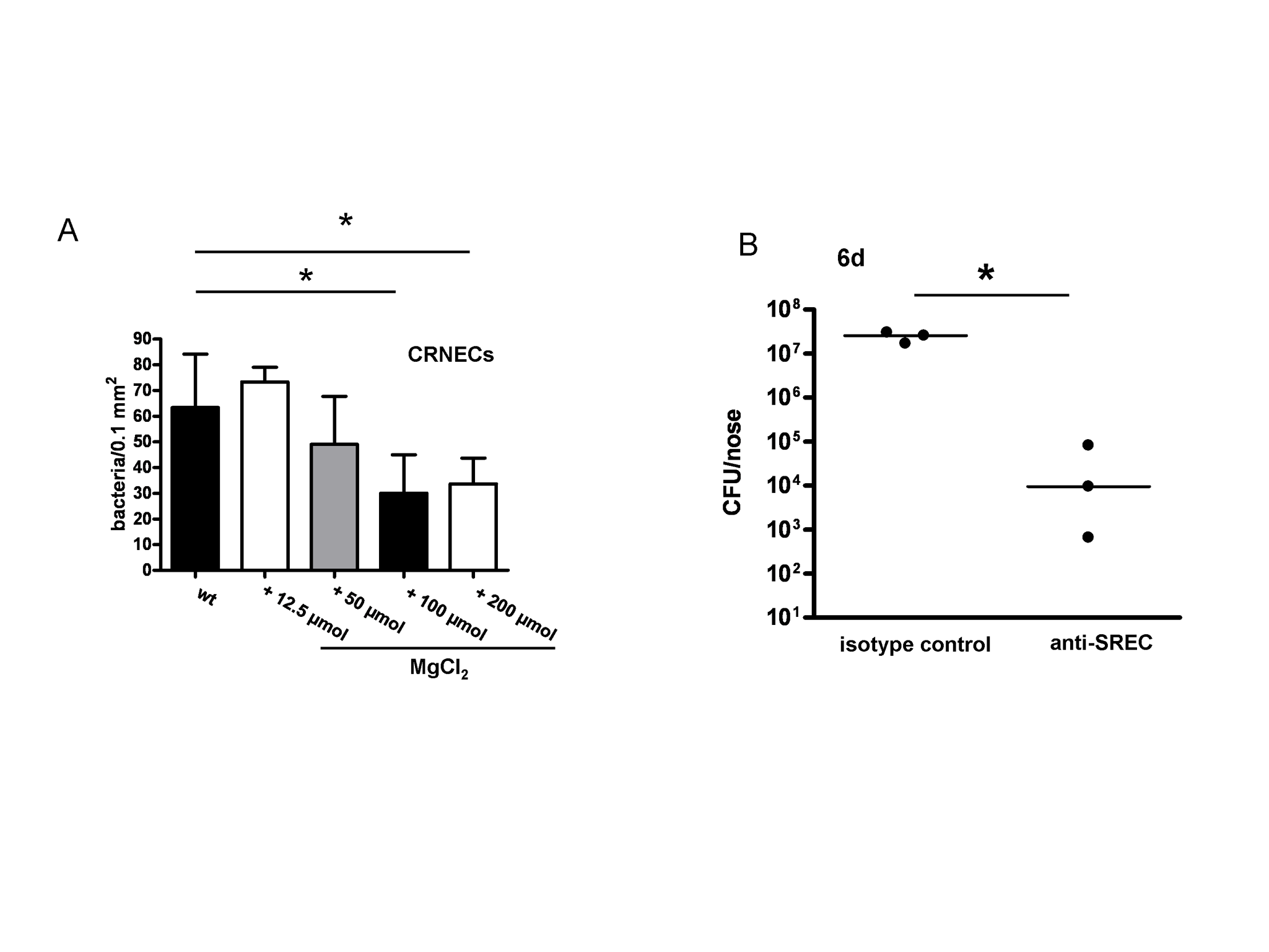

Supplement: Figure S8 — Charge dependency of WTA dependent adhesion to nasal epithelial cells and SREC-I dependent modulation of USA100 nasal colonization. Adhesion to CRNECs, under shear stress conditions in ibidi chamber slide assays, was monitored after preincubation of the bacterial inoculums with different MgCl2 concentrations. The mean and SD of 4 independent experiments are shown. Statistical analysis was performed by one-way ANOVA with Bonferroni's multiple comparison test (A). Nasal colonization in the cotton rat model was tested with USA100 (B). Bacterial numbers were determined 6 days after inoculation. 15 min prior to inoculation cotton rats were pretreated with 2 µg anti-SREC-I Fab2-fragment per nose. After 6 days the noses were dissected and the bacterial CFU was evaluated on S. aureus selective highchrome agar. Statistical analysis was performed by one-way ANOVA with Bonferroni's multiple comparison test (B). Significant differences vs. wild-type without MgCl2 are indicated by one (P<0.05), two (P<0.01), or three (P<0.001) asterisks (*). (TIF) [file ppat.1004089.s008.tif]
